# Novel coronary heart disease risk factors at 60–64 years and life course socioeconomic position: The 1946 British birth cohort

**DOI:** 10.1016/j.atherosclerosis.2014.11.011

**Published:** 2015-01

**Authors:** Rebecca Jones, Rebecca Hardy, Naveed Sattar, John E. Deanfield, Alun Hughes, Diana Kuh, Emily T. Murray, Peter H. Whincup, Claudia Thomas, NSHD Scientific and Data Collection Teams

**Affiliations:** a Population Health Research Institute, St George's, University of London, London, United Kingdom; b MRC Unit for Lifelong Health and Ageing, Institute of Epidemiology and Health Care, University College London, London, United Kingdom; c British Heart Foundation Glasgow Cardiovascular Research Centre, University of Glasgow, Glasgow, United Kingdom; d Vascular Physiology Unit, Institute of Cardiovascular Science, University College of London, United Kingdom; e National Heart and Lung Institute, Imperial College Academic Health Sciences Centre, London, United Kingdom

**Keywords:** Socioeconomic position, Life course, Inflammation, Endothelial, Adipocyte, Proinsulin, Birth cohort

## Abstract

Social disadvantage across the life course is associated with a greater risk of coronary heart disease (CHD) and with established CHD risk factors, but less is known about whether novel CHD risk factors show the same patterns. The Medical Research Council National Survey of Health and Development was used to investigate associations between occupational socioeconomic position during childhood, early adulthood and middle age and markers of inflammation (C-reactive protein, interleukin-6), endothelial function (E-selectin, tissue-plasminogen activator), adipocyte function (leptin, adiponectin) and pancreatic beta cell function (proinsulin) measured at 60–64 years. Life course models representing sensitive periods, accumulation of risk and social mobility were compared with a saturated model to ascertain the nature of the relationship between social class across the life course and each of these novel CHD risk factors. For interleukin-6 and leptin, low childhood socioeconomic position alone was associated with high risk factor levels at 60–64 years, while for C-reactive protein and proinsulin, cumulative effects of low socioeconomic position in both childhood and early adulthood were associated with higher (adverse) risk factor levels at 60–64 years. No associations were observed between socioeconomic position at any life period with either endothelial marker or adiponectin. Associations for C-reactive protein, interleukin-6, leptin and proinsulin were reduced considerably by adjustment for body mass index and, to a lesser extent, cigarette smoking. In conclusion, socioeconomic position in early life is an important determinant of several novel CHD risk factors. Body mass index may be an important mediator of these relationships.

## Highlights

• We examine associations of life course socioeconomic position (SEP) with novel coronary heart disease risk markers using novel methods to compare different life course models.
• SEP during childhood was important for IL-6 and leptin, while SEP during both childhood and early adulthood was important for CRP and proinsulin.
• BMI (but not smoking) explained a large part of these relationships.

## 1 Introduction

In the UK and other Western countries, social disadvantage across the life course is a strong determinant of coronary heart disease (CHD) risk [1–3]. Potential explanations for this socioeconomic gradient in CHD risk have centred on socioeconomic differences in the distribution of established risk factors, particularly cigarette smoking, blood pressure, blood lipids and adiposity [4,5]. These investigations have included a small number of studies that have examined the effect of socioeconomic position across the life course on established CHD risk factors, such as body mass index, blood pressure and cholesterol [6,7]. However, a substantial amount of variation in CHD is not explained by adjustment for these established risk factors [4,5].

More recently, several novel risk factors have been identified as potential predictors of increased CHD risk. These include higher levels of markers of the inflammatory response, C-reactive protein and interleukin-6, higher levels of markers of endothelial function, E-selectin and tissue plasminogen activator, the adipokine leptin and the pancreatic beta cell function marker proinsulin [8–13]. In contrast, circulating levels of the adipokine adiponectin are inversely associated with CHD risk [14,15]. There is growing interest in the possibility that these novel CHD risk factors, particularly inflammatory markers, may be mediators of the association between socioeconomic position and CHD risk [16–20]. Recent evidence suggests that markers of endothelial, adipocyte and pancreatic beta-cell function could also represent biological pathways relating socioeconomic position and CHD risk [21,22].

There is also growing evidence that low socioeconomic position in early life, as well as in adulthood, is associated with CHD risk [23]; which could be mediated through elevated circulating inflammatory markers [6,24]. Several different life course models have been advanced to define how socioeconomic position at different stages over the life course may be related to CHD risk [25]. The sensitive (or critical) period model maintains that exposure to risk during a particular stage in life (e.g. childhood) has an adverse effect on health with little or no influence of the risk factor in question outside the specified time period. The accumulation of risk hypothesis, on the other hand, proposes that the impact of the exposure is cumulative over the life course and that the longer an individual is exposed to the risk in question, the greater the adverse impact on health. Some researchers have highlighted the possible importance of social mobility [26]. However, to our knowledge no previous studies have examined which life course model best describes the associations between socioeconomic position at different stages of the life course and novel CHD risk factor patterns in middle age, when CHD risk is high.

We therefore investigated the relationship between socioeconomic position and novel CHD risk factors – particularly markers of inflammation and endothelial function, adipocyte and pancreatic beta cell function – measured at the most recent age these markers were measured (60–64 years) in participants of a British birth cohort study. Socioeconomic position was based on occupational social class assessed prospectively at three separate points during the life course (in childhood, early adulthood and middle age). We used a structured modelling approach [27] to assess whether observed associations are best explained by sensitive period, accumulation of risk or social mobility models. We also examined whether cigarette smoking and adiposity are important mediators of associations between socioeconomic position over the life course and these novel CHD risk factors.

## 2 Methods

### 2.1 Study design

The UK Medical Research Council (MRC) National Survey of Health and Development is a study of a nationally representative sample of 5362 births of all the single, legitimate births that took place in one week in March 1946 in England, Scotland and Wales [28]. Participating men and women have been followed up at regular intervals between birth and later middle age [28]. Between 2006 and 2011 (at 60–64 years of age), 2856 eligible study members (those still alive and with a known address in England, Scotland or Wales) were invited for an assessment at one of six clinical research facilities or to be visited by a research nurse at home. Full details have been reported elsewhere [27]. Invitations were not sent to those who had died (778), were living abroad (570), had previously withdrawn from the study (594) or had been lost to follow-up (564). Of those invited, 2229 (78%) were assessed: 1690 attended a clinical research facility and the remaining 539 were seen at home [29]. Physical measurements of weight and height were made and body mass index calculated; blood pressure was measured and a blood sample collected after an overnight fast.

### 2.2 Novel CHD risk factors

C-reactive protein, interleukin-6, E-selectin, tissue plasminogen activator, proinsulin, leptin and adiponectin were measured at 60–64 years of age. Blood samples were taken during the clinic or home visit and aliquots stored at −80 °C before being couriered monthly on dry ice to the Medical Research Council Human Nutrition Research laboratory in Cambridge, where C-reactive protein was analysed according to standardised protocols. Analyses of interleukin-6, E-selectin, tissue plasminogen activator, proinsulin leptin and adiponectin were undertaken by the British Heart Foundation Research Centre in Glasgow using serum and plasma aliquots stored at −70 °C. Details of assays and inter-assay coefficients of variation are provided in Supplementary Table 1. Values lower than the detection limit (1.1 mg/l for C-reactive protein, 1.0 ng/ml for E-selectin and 3.6 pmol/l for proinsulin) were assigned a notional value equal to the detection limit divided by the square root of 2 [30]. Measures of proinsulin were included in analyses only if the study member was recorded as having fasted prior to blood being taken.

### 2.3 Socioeconomic position

Measures of occupational socioeconomic position at three equally spaced time points over the life course were selected for analysis. The husband's occupation (or last job if unemployed, ill or dead) when the study member was aged 4 was chosen to represent childhood socioeconomic position, while own occupation-based socioeconomic position at ages 26 and 53 represented socioeconomic position in early adulthood and middle age respectively. Wherever possible, missing values were imputed from adjacent ages (33 values from age 11 and 14 values from age 15 for childhood socioeconomic position; 107 values from age 36 for early adulthood; 107 values from age 43 for late adulthood). Within the National Survey of Health and Development, occupational socioeconomic position is recorded using the Registrar General's six-level classification scheme [31]. For the purposes of our analyses, we re-categorised this measure into four levels: professional and intermediate (I and II); skilled non-manual (IIInm); skilled manual (IIIm); and semi-skilled and unskilled manual (IV and V).

### 2.4 Covariates

Body mass index (kg/m^2^) was calculated from height and weight measured at ages 4, 53 and 60–64 years and from self-reported height and weight at age 26. Self-reported smoking habits were used to calculate two measures of smoking: (1) current smoking at age 60–64, classified in five categories: never smoked, ex-smoker and light, medium or heavy current smoker (1–10, 11–20, or 20+ cigarettes per day respectively) and (2) Cigarette pack years, a measure of intensity and duration of smoking, estimated from number of cigarettes smoked per day at ages 20, 25, 31, 36, 43, and 53 years [32].

### 2.5 Statistical analysis

Initially, associations between socioeconomic position and the novel CHD risk factors were investigated using separate linear regression models for socioeconomic position during childhood (aged 4), early adulthood (aged 26) and middle age (aged 53). All novel risk factors were positively skewed and therefore transformed using the natural logarithm. Association between socioeconomic position and CHD risk factors did not vary markedly between men and women, therefore these analyses were performed on data on men and women combined, adjusting for sex.

We then used a structured modelling approach to select the life course models which best fitted the data for each CHD risk factor. The life course socioeconomic position models considered were as follows: (1) sensitive periods in either childhood, early adulthood or middle age; (2) accumulation of risk of low social class in childhood and early adulthood, in adulthood only, and across all three time points; and (3) upward or downward social mobility in adulthood only and social mobility between any of the three time points. To avoid zero cell counts, socioeconomic position at each time period was further collapsed into binary indicators representing manual or non-manual occupation. Model specification and constraints are described in more detail in Supplementary Table 2.

The selection of the best model has been described elsewhere [7,27]. Briefly, for each CHD risk marker, each life course model was compared to a more complex ‘saturated’ model where parameters are included for socioeconomic position at each time point, all two-way interactions and the three-way interaction, using *F* tests. If the *F* test was not statistically significant (*p* > 0.05), then there was no evidence that the more complex model explained the data better than the simpler life course model and the latter was adopted. For each CHD risk marker, the highest *p*-value for a given life course model was chosen as the best fitting model, unless the *p*-value for the ‘no effect’ model was >0.05, indicating that there was no association between socioeconomic position at any time period with that cardiac outcome.

We then fitted the identified best fitting life course model to obtain estimates of percentage differences in that specified novel CHD risk factor for the relevant difference in socioeconomic position. In order to establish whether associations between low socioeconomic position and adverse levels of novel risk markers could be explained by either adiposity or smoking, covariates representing current adiposity (body mass index at age 60–64), adiposity over the life course (body mass index at ages 4, 26 and 53), current smoking (age 60–64), and life course smoking (cigarette pack years from age 20–53) were added in turn and then simultaneously to the best-fitting life course socioeconomic position model for each novel cardio-metabolic risk factor.

All analyses were initially restricted to participants who had complete data on the risk factor in question, life course socioeconomic position trajectory and all of the covariates, but to investigate possible bias due to missing data the models were also refitted using multiple imputation. As well as the measures in the analysis models, the imputation model also included the following: birth weight; body mass index at ages 2, 6, 7, 11, 15, 20, 36 and 43 years; blood pressure at ages 36, 43, 53 and 60–64 years; number of cigarettes smoked per day at ages 20, 25, 31, 36, 43 and 53 years; E-selectin, adiponectin, triglycerides, glucose, insulin, glycated hemoglobin (HbA1c), cholesterol, high-density lipoprotein-cholesterol and waist-to-hip ratio at age 60–64 years; and response at the 2006–2010 data collection. Participants who died prior to or during the 2006–2010 data collection were excluded from the imputation process. Fifty imputed datasets were obtained via chained equations using 50 cycles per dataset [32]. All analyses were performed using Stata 12 (StataCorp 2011). Sensitivity analyses were also conducted to examine the effects of using socioeconomic position at different ages (replacing age 4 with age 11 years, age 26 with age 36 years and age 53 with 43 years).

## 3 Results

Of the 2229 participants, 2077 (93.2%) with data available for at least one novel CHD risk factor and a measure of socioeconomic position for at least one of the three time periods, were included in the initial analyses. A summary of the novel CHD risk factors at age 60–64 years and socioeconomic position, body mass index and smoking over the life course for these participants are presented in Table 1.

### 3.1 Associations between socioeconomic position and the novel CHD risk factors at different stages of the life course

We found strong evidence for associations between socioeconomic position and six of the seven novel CHD risk factors; individuals in lower socioeconomic position displayed more adverse levels of C-reactive protein, interleukin-6, E-selectin, proinsulin, leptin and adiponectin (Table 2). The inflammatory markers C-reactive protein and interleukin-6 were both associated with socioeconomic position in childhood, early adulthood and middle age. Leptin and proinsulin were associated with socioeconomic position in childhood and early adulthood, though less clearly in middle-age; E-selectin and adiponectin were associated with childhood but not adult socioeconomic position. Socioeconomic position showed no association with tissue plasminogen activator at any stage of the life course. The associations observed were similar in men and women with no consistent evidence for interactions involving sex (data not shown).

### 3.2 Comparison of life course models

In order to compare model fit of the different life course models, the sample was restricted further to cohort members who had measures of socioeconomic position at all three time points (*n* = 1840, 88.6%). Details of participant numbers and mean levels of the CHD risk factors for each of the eight possible trajectories of socioeconomic position are presented in Supplementary Table 3, while the results of model fitting are presented in Table 3. The childhood sensitive period model provided the best model fit for both interleukin-6 and leptin (*p*-values: 0.856, 0.936 respectively), while the childhood and early adult accumulation model offered the best fit for C-reactive protein and proinsulin (*p* values: 0.701, 0.360) (Table 3). For E-selectin, tissue plasminogen activator and adiponectin, the ‘no effect’ model provided the best fit and regression estimates were non-significant for all life course models; consequently they were omitted from further analyses.

Details of the selected life course models for C-reactive protein, interleukin-6, proinsulin and leptin, controlling for sex, are presented in Table 4. Interleukin-6 and leptin were respectively 12.2% (95% CI: 4.0–20.4) higher and 16.7% (95% CI: 7.6–25.9) higher among individuals with manual compared to those with non-manual childhood socioeconomic position after adjustment for sex. C-reactive protein increased by 16.3% (95% CI: 9.6–23.0) and proinsulin by 10.7% (95% CI: 5.3–16.1) for each unit increase in early life socioeconomic position score (on a scale from 0 to 2, with 2 indicating manual socioeconomic position in both childhood and early adulthood).

### 3.3 Adjustment for adiposity and cigarette smoking: sensitivity analyses

The effects of adjustment for current and life course body mass index and cigarette smoking of these associations between socioeconomic position and novel CHD risk factors are shown in Table 4. Adjustment for current and life course body mass index attenuated the associations observed between socioeconomic position and four CHD risk factors (C-reactive protein, interleukin-6, proinsulin and leptin); only the association between socioeconomic position and C-reactive protein remaining statistically significant (Table 4). Attenuation was similar, but less marked, when adjusting for current and life course smoking, with the exception of associations with proinsulin and leptin which were slightly strengthened by adjustment for current smoking status. Additional adjustment for systolic blood pressure, diastolic blood pressure and LDL cholesterol, both singly and simultaneously, had no material effects on the Results (data not shown), so were not included in the analysis.

Sensitivity analyses were carried out using alternate age-points for childhood socioeconomic position (11 rather than 4 years), young adulthood (36 rather than 26 years) and middle age (43 rather than 53 years); these changes did not materially affect the results or conclusions. Results using multiple imputation were similar to those using complete case analysis (Supplementary Table 4), except that associations between socioeconomic position, interleukin-6 and proinsulin were still apparent after adjustment for current and life course body mass index.

## 4 Discussion

### 4.1 Main findings

In this prospective birth cohort study, and using novel analytic approaches, we observed that low socioeconomic position in childhood was associated with higher values of several novel CHD risk factors, including C-reactive protein, interleukin-6, leptin and proinsulin at 60–64 years. In addition, socioeconomic position in young adulthood was also important for C-reactive protein and proinsulin, suggesting the possibility of cumulative effects operating across childhood and early adult life. These associations were attenuated considerably by adjustment for body mass index and, to a lesser extent cigarette smoking, suggesting that body mass index in particular may be an important mediator of the relationship between socioeconomic position and these novel CHD risk factors.

### 4.2 Comparison with previous findings

Several studies have reported associations between low socioeconomic position and higher levels of inflammatory and endothelial function markers such as C-reactive protein, interleukin-6 and tissue plasminogen activator, which are consistent with those reported in the present study [16–18,34]. However, most previous studies investigated socioeconomic position at a single time point, usually in adult life; few have investigated different life course models such as sensitive period or accumulation of risk models [6,16,33]; those which have did not attempt to distinguish between them or to identify a best fitting model. Both the Framingham Offspring Study and the 1958 British Birth Cohort reported that lower cumulative socioeconomic position over the life course was related to higher levels of C-reactive protein [6,35], as in the present study. The former study showed that participants with low cumulative socioeconomic position scores (father's education, own education and own occupation) had higher C-reactive protein scores in adulthood. The latter study showed that after mutual adjustment for socioeconomic position at each of the other time points only associations with childhood and early adult socioeconomic position remained. Our results extend these earlier reports by showing that the childhood and early adult accumulation model displayed the best model fit, while also showing that life course accumulation models (both the childhood and early adulthood only and the model with all three time points), explained the C-reactive protein data as well as the saturated model. An earlier report from the Young Finns Study [16] however found only a weak association between parental socioeconomic position with C-reactive protein levels; this may reflect the early age at outcome (24–39 years), substantially earlier than in the present report.

As in our study, the Framingham Offspring Study found associations between childhood socioeconomic position and interleukin-6 [6]; again, the present report extends this earlier observation by showing that the childhood sensitive period model was actually the best fitting model for interleukin-6. However, our observation that socioeconomic position at any point in the life course was not related to tissue plasminogen activator contrasts with a report from the 1958 British Birth Cohort showing that childhood but not adult socioeconomic position was related to tissue plasminogen activator [35]. While the difference from our findings may reflect the substantially larger size and greater statistical power of the 1958 Cohort, our finding of a null association between socioeconomic position and E-selectin, another key endothelial function marker, supports the validity of our observation.

To our knowledge, this is the first report on associations between life course socioeconomic position and proinsulin, leptin and adiponectin. The presence of an association between childhood socioeconomic position and leptin, rather than adiponectin, may well reflect its stronger association with body fatness [36], which shows a strong association with socioeconomic position from childhood [37]. This would also be consistent with our finding that adjustment for body mass index substantially reduced the associations between socioeconomic position and C-reactive protein, interleukin-6, proinsulin and leptin, emphasizing an important mediating role of body fatness in the association between socioeconomic position over the life course and novel CHD risk factors in later life; consistent with observations in the 1958 British Birth Cohort [35], and The Framingham Offspring Study [19].

### 4.3 Strengths and limitations

A major strength of this study is the availability of data on several novel CHD risk factors representing several biological pathways (inflammation, endothelial, adipocyte and pancreatic beta function), measured in late middle age in a large birth cohort with detailed historical records, permitting us to investigate the role of socioeconomic position at different time points over the life course. The structured modelling approach we used to compare several different life course socioeconomic position models is an improvement over traditional regression models where results are interpreted from a single pre-specified model without considering the merits of alternative models [7]. Although the approach has limitations, including the requirement to merge the four socioeconomic position categories into two and to include not more than three age-groups, it does provide an indication of best fitting life course exposure patterns, although in some cases the distinction between the best fitting and the next best fitting models is not strong. However, in previous analyses, the results yielded by this method matched socioeconomic trends seen in models with four categories [7]. In addition, results were not materially affected by the use of alternative age-points for socioeconomic position were used, indicating that findings are likely to be robust to the inclusion of socioeconomic position at other ages. Although the analyses presented are entirely based on the occupation of the male partner, the present cohort was characterized by high rates of paternal employment and by low rates of maternal employment, and by low rates of separation and divorce, suggesting that the male occupational measure is likely to have been particularly appropriate in this context.

Although the original birth cohort on which this investigation was based was highly representative of the British population [29], a relatively large proportion of our sample did not have complete data (particularly on potential mediators of the relationship between socioeconomic position and CHD risk), raising the possibility of selection bias. However, with detailed information on participant characteristics from earlier phases of measurement, it was possible to carry out robust multiple imputation analyses, specifically to examine the impact of selection bias on the results observed. These analyses did not materially affect the main results, but suggested that the strength of the associations between socioeconomic position and these novel CHD risk factors, interleukin-6 in particular, were slightly underestimated in the subsample with complete data.

### 4.4 Implications

The results suggest that childhood socioeconomic position, acting either as a sensitive period effect or as part of a cumulative life course model, is associated with several novel CHD risk factors in later middle age. If the associations between these novel risk factors and CHD are shown to be causal, as appears increasingly likely for interleukin-6 [38] though not for C-reactive protein [39], this would add weight to earlier suggestions that reducing socioeconomic inequalities in childhood could be particularly important for the prevention of CHD in later life [40]. The strength of the associations between socioeconomic position and CHD risk factors, even if causal, would be too small to be clinically important. For example, the increase in leptin level associated with being in a manual rather than a non-manual social class group (∼20%) would be associated with an increase in the relative risk of coronary heart disease of ∼1–2% [12] at the individual level. However, such an increase, if affecting the half of the study population in manual occupations, could be of appreciable public health importance.

A second potentially important implication is the role played by adult body fatness as a mediator of the associations between childhood socioeconomic position and several novel CHD risk factors, particularly C-reactive protein, interleukin-6, proinsulin and leptin. This suggests that targeted prevention and treatment of overweight and obesity in low socioeconomic groups, particularly at a population level and throughout the life course, could play a particularly important role in reducing socioeconomic inequalities in CHD in adult life.

## Funding

This work was supported by the Medical Research Council [U1200632239, MC_UU_12019/1, MC_UU_12019/2 and G1001143].

## Disclosure of conflict of interests

None.

## Figures and Tables

**Table 1:** Summary statistics of the sample, National Survey of Health and Development 1946–2011.

	*N*	All (*N* = 2077)	Men (*N* = 1010)	Women (*N* = 1067)	*p* Value gender difference
**Novel coronary heart disease risk factors (age 60–64) – geometric mean (SD)**					
C-Reactive protein (mg/l)	2063	2.31 (2.43)	2.24 (2.46)	2.39 (2.41)	0.087
Interleukin-6 (pg/ml)	2049	2.08 (2.03)	2.15 (2.02)	2.01 (2.05)	0.033
E-selectin (ng/ml)	2049	35.35 (1.57)	36.81 (1.56)	34.02 (1.57)	<0.001
Tissue-plasminogen activator (ng/ml)	1795	8.51 (1.85)	9.09 (1.84)	7.98 (1.85)	<0.001
Proinsulin (pmol/l)	1795	8.82 (2.03)	10.06 (2.08)	7.78 (1.93)	<0.001
Leptin (ng/ml)	2053	12.56 (2.56)	7.53 (2.16)	20.35 (2.29)	<0.001
Adiponectin (μg/ml)	2051	11.77 (2.06)	8.55 (2.00)	15.91 (1.85)	<0.001
Socioeconomic position^a^**– *N* (%)**					
Childhood (age 4)					
I and II	1975	508 (25.7)	247 (25.5)	261 (25.9)	0.93
IIInm		396 (20.0)	189 (19.5)	207 (20.5)	
IIIm		571 (28.9)	283 (29.2)	288 (28.6)	
IV and V		500 (25.3)	248 (25.6)	252 (25.0)	
Early adulthood (age 26)					
I and II	1981	689 (34.8)	392 (40.7)	297 (29.1)	<0.001
IIInm		624 (31.5)	145 (15.1)	479 (47.0)	
IIIm		391 (19.7)	304 (31.6)	87 (8.5)	
IV and V		277 (14.0)	121 (12.6)	156 (15.3)	
Middle age (age 53)					
I and II	2000	962 (48.1)	556 (56.6)	406 (39.9)	<0.001
IIInm		467 (23.4)	103 (10.5)	364 (35.8)	
IIIm		306 (15.3)	234 (23.8)	72 (7.1)	
IV and V		265 (13.3)	90 (9.2)	175 (17.2)	
**Body mass index –mean (SE)**					
Age 4 (kg/m^2^)	1799	16.18 (1.64)	16.31 (1.67)	16.06 (1.61)	0.0014
Age 26 (kg/m^2^)	1821	22.67 (2.91)	23.19 (2.73)	22.19 (2.99)	0.0000
Age 53 (kg/m^2^)	1965	27.13 (4.51)	27.24 (3.75)	27.02 (5.11)	0.2959
Age 60–64 (kg/m^2^)	2071	27.86 (4.88)	27.91 (4.10)	27.80 (5.51)	0.6153
**Cigarette smoking**					
Pack years to age 53– mean (SE)	1660	9.99 (0.33)	11.54 (0.50)	8.49 (0.42)	0.0000
At age 60–64 – *N* (%)					
Never smoked	1889	911 (48.2)	385 (42.0)	526 (54.1)	<0.001
Ex-smoker		773 (40.9)	434 (47.4)	339 (34.8)	
Light current smoker		72 (3.8)	31 (3.4)	41 (4.2)	
Moderate current smoker		99 (5.2)	42 (4.6)	57 (5.9)	
Heavy current smoker		34 (1.8)	24 (2.6)	10 (1.0)	

a Socioeconomic position: I and II = professional and intermediate; IIInm = skilled non-manual; IIIm = skilled manual; IV and V = partly skilled or unskilled.

**Table 2:** Sex and age-adjusted^a^ percentage differences (95% CI) in novel coronary heart disease (CHD) risk markers for lower socioeconomic position relative to the highest position (reference group).^b^

	Inflammatory and endothelial markers				Pancreatic and adiposity markers		
	C-reactive protein	Interleukin-6	E-selectin	Tissue plasminogen activator	Proinsulin	Leptin	Adiponectin
	(*N* = 2063)	(*N* = 2049)	(*N* = 2049)	(*N* = 1795)	(*N* = 1795)	(*N* = 2053)	(*N* = 2051)
**Childhood**							
I and II	–	–	–	–	–	–	–
IIInm	3.2	7.9	7.1	4.0	3.4	12.4	−9.5
	(−8.5 to 14.8)	(−1.4 to 17.3)	(1.2–13.0)	(−4.6 to 12.5)	(−6.2 to 13.0)	(1.9–22.9)	(−18.1 to −0.9)
IIIm	22.6	20.5	7.1	6.8	19.9	22.6	−11.8
	(12.0–33.2)	(12.0–29.0)	(1.8–12.5)	(−1.0 to 14.6)	(11.1–28.7)	(13.1–32.2)	(−19.7 to −3.9)
IV and V	25.1	17.6	8.0	4.9	19.1	21.9	−10.9
	(14.2–36.1)	(8.8–26.4)	(2.4–13.5)	(−3.3 to 13.0)	(9.9–28.3)	(12.0–31.8)	(−19.0 to −2.7)
*Trend p*	<*0.001*	<*0.001*	*0.002*	*0.171*	<*0.001*	<*0.001*	*0.013*
**Early adulthood**							
I and II	–	–	–	–	–	–	–
IIInm	13.8	8.9	−1.7	−2.0	4.1	16.2	−2.6
	(3.8–23.8)	(0.8–16.9)	(−6.7 to 3.4)	(−9.3 to 5.4)	(−4.2 to 12.4)	(7.2–25.2)	(−10.0 to 4.9)
IIIm	16.2	7.7	1.7	5.7	17.8	12.3	−9.8
	(5.1–27.3)	(−1.3 to 16.7)	(−4.0 to 7.3)	(−2.6 to 14.0)	(8.5–27.2)	(2.2–22.4)	(−18.1 to −1.6)
IV and V	36.2	22.3	5.4	2.5	16.8	13.5	−3.1
	(23.8–48.7)	(12.3–32.3)	(−1.0 to 11.7)	(−6.8 to 11.8)	(6.2–27.3)	(2.3–24.8)	(−12.3 to 6.2)
*Trend p*	<*0.001*	<*0.001*	*0.104*	*0.271*	<*0.001*	*0.007*	*0.155*
**Middle age**							
I and II	–	–	–	–	–	–	–
IIInm	16.0	14.1	−0.9	1.8	4.9	15.6	0.7
	(5.7–26.2)	(5.9–22.3)	(−6.1 to 4.3)	(−5.7 to 9.4)	(−3.7 to 13.4)	(5.4–26.8)	(−6.9 to 8.3)
IIIm	11.0	6.3	1.4	2.1	6.5	9	−0.4
	(−0.5 to 22.5)	(−2.9 to 15.6)	(−4.4 to 7.3)	(−6.6 to 10.7)	(−3.3 to 16.3)	(−1.8 to 20.9)	(−8.9 to 8.2)
IV and V	26.6	15.2	1.2	−7.6	7.6	12.7	−3.0
	(14.4–38.8)	(5.4–25.1)	(−5.1 to 7.4)	(−16.9 to 1.6)	(−2.9 to 18.1)	(0.9–25.9)	(−12.2 to 6.1)
*Trend p*	<*0.001*	*0.002*	*0.579*	*0.355*	*0.035*	*0.090*	*0.386*

a Age at blood draw (60–64 years).

b Socioeconomic position: I and II = professional and intermediate; IIInm = skilled non-manual; IIIm = skilled manual; IV and V = partly skilled or unskilled.

**Table 3:** *P*-values from partial *F* tests comparing each life course model relating socioeconomic position and CHD risk markers with the saturated model.^a^

Life course social class model	Inflammatory and endothelial markers				Pancreatic and adiposity markers		
	C-reactive protein (*N* = 1827)	Interleukin-6 (*N* = 1818)	E-selectin (*N* = 1818)	Tissue plasminogen activator (*N* = 1601)	Proinsulin (*N* = 1601)	Leptin (*N* = 1822)	Adiponectin (*N* = 1820)
No effect	0.000	0.001	**0.314**	**0.220**	0.000	0.010	**0.313**
**Sensitive period models**							
Childhood (age 4)	**0.125**	**0.856**	**0.684**	**0.259**	**0.115**	**0.936**	**0.723**
Early adulthood (age 26)	0.003	0.008	**0.556**	**0.288**	0.001	0.011	**0.473**
Middle age (age 53)	0.000	0.003	**0.259**	**0.218**	0.000	0.012	**0.256**
**Accumulation models**							
Childhood and early adulthood	**0.701**	**0.625**	**0.868**	**0.359**	**0.297**	**0.352**	**0.830**
Early adulthood and middle age	0.001	0.009	**0.414**	**0.151**	0.000	0.015	**0.376**
Whole life	**0.163**	**0.258**	**0.666**	**0.182**	0.006	**0.184**	**0.632**
**Social mobility models**							
Adulthood (age 26 & 53)	0.000	0.001	**0.260**	**0.339**	0.000	0.006	**0.251**
Whole life	0.000	0.006	**0.295**	**0.316**	0.000	**0.085**	**0.554**

Bold indicates p-value > 0.05.

a Larger *p* values represent better model fit. Shaded cells indicate the selected model – the most parsimonious model with a good fit to the data.

**Table 4:** Selected life course social class model for each novel coronary heart disease (CHD) risk marker adjusted for sex, age a and potential mediators.

	Model 1	Model 2	Model 3	Model 4	Model 5	Model 6
	Sex and age^a^ only	Current smoking (age 60–64)	Life course smoking (ages 20–53)	Current body mass index (age 60–64)	Life course body mass index (ages 4, 26 and 53)	All
**Childhood sensitive period model (manual vs non-manual social class in childhood)**						
Interleukin-6 (*N* = 1144)	12.2	9.3	8.8	7.1	8.4	3.0
	(4.0–20.4)	(1.0–17.5)	(0.5–17.0)	(−1.0 to 15.2)	(0.2–16.6)	(−5.1 to 11.1)
Leptin (*N* = 1147)	16.7	18.4	15.6	0.6	4.8	2.4
	(7.6–25.9)	(9.2–27.6)	(6.4–24.9)	(−6.4 to 7.6)	(−3.0 to 12.5)	(−4.6 to 9.4)
**Childhood + early adulthood accumulation model (each additional time in manual vs non-manual social class)**						
C-reactive protein (*N* = 1151)	16.3	13.8	13.6	11.3	13.5	8.2
	(9.6–23.0)	(6.9–20.6)	(6.7–20.5)	(4.7–17.8)	(6.8–20.2)	(1.4–15.0)
E-selectin (*N* = 1144)	2.4	2.4	1.5	0.3	1.0	0.0
	(−1.0 to 5.8)	(−1.1 to 5.9)	(−1.9 to 5.0)	(−3.0 to 3.7)	(−2.4 to 4.4)	(−3.5 to 3.5)
Proinsulin (*N* = 1018)	10.7	11.4	8.7	3.8	6.0	3.9
	(5.3–16.1)	(5.9–17.0)	(3.2–14.2)	(−1.0 to 8.6)	(1.1–11.0)	(−1.0 to 8.8)

a Age at blood draw (60–64 years).
