# Supplementary material for: Novel coronary heart disease risk factors at 60–64 years and life course socioeconomic position: The 1946 British birth cohort
Source: Atherosclerosis. 2015 Jan;238(1):70–6. doi: 10.1016/j.atherosclerosis.2014.11.011 (PMC4286122; doi:10.1016/j.atherosclerosis.2014.11.011)
Supplement: Supplementary file 1 [file mmc1.docx]

**Supplementary Table 1: Methods and inter-assay coefficients of variation (CV) for cardiometabolic risk factor assessed from blood samples.**

| **Risk factor** | **Units** | **Assay/Method** | **CV (%)** |
| --- | --- | --- | --- |
| C-reactive protein (CRP) | mg/l | Particle-enhanced immunoturbidimetric assay | 4.3% at 3.4 mg/L 1.8% at 11.9 mg/L |
| Interleukin-6 (IL6) | pg/ml | Enzyme-linked immunosorbent assay (ELISA) | 6.5% |
| E-selectin | ng/ml | High sensitivity ELISA | <10.0% |
| Tissue plasminogen activator (tPA) | ng/ml | Enzyme-linked immunosorbent assay (ELISA) | 6.6% |
| Proinsulin | Pmol/L | Enzyme-linked immunosorbent assay (ELISA) | <5.0% |
| Leptin | ng/ml | In-house radioimmunoassay validated against commercially available assays | <10.0% |
| Adiponectin | ug/ml | Enzyme-linked immunosorbent assay (ELISA) | <7.5% |

**Supplementary Table 2: Model specification and constraints for given life course models**

|  | **Life-course model specification** | **Constraints** |
| --- | --- | --- |
| Saturated model | α + b_1_S_1_ + b_2_S_2_ + b_3_S_3_ + θ_12_S_1_S_2_+ θ_13_ S_1_S_3_ + θ_23_S_2_S_3_+ θ_123_S_1_S_2_S_3_ |  |
| No effect | α |  |
| **Sensitive period models** |  |  |
| Childhood (age 4) | α + b_1_S_1_ |  |
| Early adulthood (age 26) | α + b_2_S_2_ |  |
| Middle age (age 53) | α + b_3_S_3_ |  |
| **Accumulation models** |  |  |
| Childhood and early adulthood | α + S_1_ (b_1_ + b_2_) |  |
| Early adulthood and middle age | α + S_1_ (b_2_ + b_3_) |  |
| Whole life | α + S_1_ (b_1_ + b_2_ + b_3_) |  |
| **Social mobility models** |  |  |
| Adulthood (age 26 to 53) | α + b_2_S_2_ + b_3_S_3_ + θ_23_S_2_S_3_ | θ_23_ S_2_S_3_ = - (b_2_ + b_3_) |
| Whole life | α + b_1_S_1_ + b_2_S_2_ + b_3_S_3_ + θ_12_S_1_S_2_+ θ_23_ S_2_S_3_ | b_2_ = (b_1_ + b_3_), & θ_12_ = θ_23_ = - b_2_ |

For the sensitive period models, a score of 1 indicates a manual socioeconomic position. In the childhood and early adult accumulation model, a score of 2 (on a scale from 0-2) indicated manual socioeconomic position both in childhood and early adulthood; in the adult accumulation model, a score of 2 (again on a scale from 0-2) indicated manual socioeconomic position both in early adulthood and middle age; and in the whole life accumulation model a score of 3 (on a scale from 0-3) indicated manual socioeconomic position at all three time points. Adult social mobility was modelled by fitting socioeconomic position in early adulthood and middle age and their interaction, with those individuals with the same socioeconomic position at both time periods constrained to have the same mean outcomes. The more general social mobility model was specified in a similar way, but utilising socioeconomic position indicators for all three time points.

**Supplementary table 3: Geometric means (standard errors) of cardiometabolic risk factor by social class trajectory over the life course**

| **Life course  social class ^a^** | | | **N (%)** | **Inflammatory and endothelial markers** | | | | **Pancreatic and adiposity markers** | | |
| --- | --- | --- | --- | --- | --- | --- | --- | --- | --- | --- |
| **Child- hood** ^b^ | **Early Adult** ^c^ | **Middle Age** ^d^ |  | **CRP** (mg/l) | **IL-6** (pg/ml) | **E-Selectin** (ng/ml) | **t-PA** (ng/ml) | **Proinsulin** (pmol/l) | **Leptin** (ng/ml) | **Adiponectin** (ug/ml) |
| **Men** | | | | | | | | | | |
| 0 | 0 | 0 | 305 (33.6) | 2.04 (0.11) | 2.00 (0.08) | 35.0 (0.9) | 0.34 (0.34) | 8.5 (0.3) | 6.9 (0.3) | 8.8 (0.3) |
| 0 | 0 | 1 | 19 (2.1) | 1.77 (0.30) | 2.21 (0.35) | 35.4 (5.9) | 1.89 (1.89) | 9.7 (1.7) | 8.6 (1.7) | 10.6 (1.6) |
| 0 | 1 | 0 | 43 (4.7) | 2.20 (0.31) | 1.98 (0.19) | 39.3 (2.5) | 0.57 (0.57) | 11.7 (1.7) | 7.6 (0.9) | 7.1 (0.7) |
| 0 | 1 | 1 | 41 (4.5) | 2.11 (0.33) | 1.92 (0.20) | 35.8 (2.6) | 0.92 (0.92) | 8.6 (1.1) | 7.1 (0.9) | 8.8 (1.2) |
| 1 | 0 | 0 | 167 (18.4) | 2.12 (0.14) | 2.10 (0.12) | 37.0 (1.3) | 0.41 (0.41) | 10.6 (0.6) | 8.0 (0.5) | 8.4 (0.4) |
| 1 | 0 | 1 | 23 (2.5) | 2.45 (0.51) | 2.78 (0.51) | 41.4 (4.5) | 1.88 (1.88) | 8.7 (1.2) | 8.8 (1.4) | 8.4 (1.4) |
| 1 | 1 | 0 | 98 (10.8) | 2.84 (0.24) | 2.57 (0.17) | 40.2 (1.9) | 0.53 (0.53) | 12.9 (1.1) | 8.4 (0.6) | 8.3 (0.5) |
| 1 | 1 | 1 | 213 (23.4) | 2.41 (0.15) | 2.27 (0.12) | 37.6 (1.1) | 0.40 (0.40) | 10.0 (0.5) | 7.4 (0.4) | 8.7 (0.5) |
|  | **Total** |  | **909** | **2.24 (0.06)** | **2.15 (0.05)** | **36.7 (0.5)** | **0.19 (0.19)** | **9.8 (0.2)** | **7.5 (0.2)** | **8.5 (0.2)** |
| **Women** | | | | | | | | | | |
| 0 | 0 | 0 | 336 (36.1) | 1.99 (0.08) | 1.81 (0.07) | 33.2 (0.8) | 0.26 (0.26) | 7.1 (0.3) | 18.2 (0.8) | 17.2 (0.6) |
| 0 | 0 | 1 | 41 (4.4) | 2.60 (0.39) | 1.76 (0.18) | 35.9 (2.8) | 1.01 (1.01) | 6.8 (0.7) | 19.4 (2.5) | 16.7 (1.7) |
| 0 | 1 | 0 | 40 (4.3) | 1.98 (0.34) | 1.94 (0.26) | 33.7 (2.2) | 0.63 (0.63) | 6.8 (0.6) | 17.6 (2.6) | 17.4 (1.7) |
| 0 | 1 | 1 | 14 (1.5) | 2.23 (0.40) | 1.74 (0.33) | 34.5 (3.0) | 0.80 (0.80) | 7.0 (1.3) | 19.7 (2.9) | 12.1 (1.8) |
| 1 | 0 | 0 | 273 (29.3) | 2.59 (0.14) | 2.12 (0.09) | 34.9 (0.9) | 0.34 (0.34) | 7.9 (0.3) | 21.9 (1.1) | 15.6 (0.6) |
| 1 | 0 | 1 | 67 (7.2) | 2.33 (0.20) | 2.07 (0.18) | 31.0 (1.8) | 0.74 (0.74) | 8.3 (0.6) | 22.3 (2.2) | 14.9 (1.3) |
| 1 | 1 | 0 | 57 (6.1) | 2.97 (0.39) | 2.02 (0.17) | 32.5 (3.0) | 0.79 (0.79) | 8.5 (0.7) | 21.7 (2.3) | 14.8 (1.3) |
| 1 | 1 | 1 | 103 (11.1) | 3.21 (0.30) | 2.30 (0.16) | 35.5 (1.7) | 0.54 (0.54) | 9.1 (0.8) | 23.9 (1.9) | 14.7 (1.0) |
|  | **Total** |  | **931** | **2.39 (0.06)** | **2.01 (0.04)** | **34.0 (0.5)** | **0.16 (0.16)** | **7.6 (0.2)** | **20.4 (0.5)** | **15.9 (0.3)** |

**^a^** Social class: 1 = manual; 0 = non-manual; ^b^ childhood = father’s social class at age 4 (or 11 or 15); ^c^ early adulthood = own social class at age 26 (or 36); ^d^ middle age = own social class at age 53 (or 43).

**Supplementary Table 4: Selected life course social class model for each cardiometabolic risk factor adjusted for potential mediators ^a^ after multiple imputation** ^b^

|  | **Model 1** | **Model 2** | **Model 3** | **Model 4** | **Model 5** | **Model 6** |
| --- | --- | --- | --- | --- | --- | --- |
|  | **Basic** | **Current Smoking** | **Life Course Smoking ^b^** | **Current BMI** | **Life Course BMI ^c^** | **All ^d^** |
| **C-Reactive Protein** – Childhood + early adulthood accumulation model **^e^** (N=2,065) | | | | | | |
| Each additional time in manual vs non-manual social class | 17.3 | 14.4 | 14.3 | 12.2 | 13.8 | 8.9 |
|  | (11.6 to 23.1) | (8.8 to 20.2) | (8.7 to 20.2) | (7.0 to 17.7) | (8.4 to 19.5) | (3.7 to 14.5) |
| **Interleukin 6** – Childhood sensitive period model **^f^** (N=2,052) | | | | | | |
| Manual vs non-manual social class in childhood | 16.8 | 13.0 | 13.2 | 11.1 | 12.0 | 6.5 |
|  | (9.7 to 24.3) | (6.2 to 20.3) | (6.4 to 20.4) | (4.5 to 18.2) | (5.3 to 19.2) | (0.1 to 13.2) |
| **Proinsulin** – Childhood + early adulthood accumulation model **^e^** (N=1,727) | | | | | | |
| Each additional time in manual vs non-manual social class | 12.0 | 12.3 | 9.7 | 4.6 | 6.8 | 4.5 |
|  | (7.5 to 16.6) | (7.7 to 17.1) | (5.3 to 14.4) | (0.9 to 8.4) | (2.9 to 10.8) | (0.7 to 8.4) |
| **Leptin** – Childhood sensitive period model **^f^** (N=2,056) | | | | | | |
| Manual vs non-manual social class in childhood | 18.3 | 20.1 | 17.2 | -0.1 | 4.8 | 1.2 |
|  | (10.2 to 27.1) | (11.8 to 29.0) | (9.0 to 25.9) | (-5.3 to 5.3) | (-1.4 to 11.4) | (-4.0 to 6.8) |

**^a^** All analyses are adjusted for sex.

^b^ As well as the measures in the analysis models (CRP, IL 6, proinsulin and leptin; social class in childhood, early adulthood and middle age; BMI at ages 4, 26, 53 and 60 64; current smoking and cigarette pack years), the imputation model also included the following: birth weight; BMI at ages 2, 6, 7, 11, 15, 20, 36 and 43 years; blood pressure at ages 36, 43, 53 and 60 64 years; number of cigarettes smoked per day at ages 20, 25, 31, 36, 43 and 53 years; E selectin, t PA, adiponectin, triglycerides, glucose, insulin, HbA1c, LDL cholesterol, HDL cholesterol and waist-to-hip ratio at age 60 64 years; and response at the 2006 2010 data collection. Participants who died prior to or during the 2006 2010 data collection were excluded from the imputation process. Fifty imputed datasets were obtained via chained equations using 50 cycles per dataset.

^c^ Life course smoking = cigarette pack years from age 20-53.

^d^ Life course BMI = BMI at ages 4, 26 and 53.

^e^ All = current and life course smoking and BMI.

^f^ For the early life accumulation model, statistics are percentage increase (95% CI) in the cardiometabolic risk factor for each additional time point spent in a manual social class during childhood and early adulthood.

^g^ For the childhood critical period model, statistics are percentage differences (95% CI) in the cardiometabolic risk factor for manual social class relative to non-manual social class in childhood.
